# Actin cytoskeleton remodeling disrupts physical barriers to infection and presents entry receptors to respiratory syncytial virus

**DOI:** 10.1099/jgv.0.001923

**Published:** 2023-11-28

**Authors:** Quinten J. Kieser, Madison J. Granoski, Ryley D. McClelland, Cameron Griffiths, Leanne M. Bilawchuk, Aleksandra Stojic, Farah Elawar, Kyla Jamieson, David Proud, David J. Marchant

**Affiliations:** ^1^​ Department of Medical Microbiology and Immunology, University of Alberta, Edmonton, T6G-2E1, Canada; ^2^​ Department of Biomedical Engineering, University of Virginia, Charlottesville, VA 22908,, USA; ^3^​ Department of Physiology and Pharmacology, Snyder Institute for Chronic Diseases, Cumming School of Medicine, University of Calgary, Calgary, Alberta T2N 4Z6, Canada

**Keywords:** actin cytoskeleton, IGF1R, PI3 kinase, receptor, RSV

## Abstract

RSV is the leading cause of infant hospitalizations and a significant cause of paediatric and geriatric morbidity worldwide. Recently, we reported that insulin-like growth factor 1 receptor (IGF1R) was a receptor for respiratory syncytial virus (RSV) in airway epithelial cells and that activation of IGF1R recruited the coreceptor, nucleolin (NCL), to the cell surface. Cilia and mucus that line the airways pose a significant barrier to viral and bacterial infection. The cortical actin cytoskeleton has been shown by others to mediate RSV entry, so we studied the roles of the RSV receptors and actin remodelling during virus entry. We found that IGF1R expression and phosphorylation were associated with the ability of RSV to infect cells. Confocal immunofluorescence imaging showed that actin projections, a hallmark of macropinocytosis, formed around viral particles 30 min after infection. Consistent with prior reports we also found that virus particles were internalized into early endosome antigen-1 positive endosomes within 90 min. Inhibiting actin polymerization significantly reduced viral titre by approximately ten-fold. Inhibiting PI3 kinase and PKCζ in stratified air-liquid interface (ALI) models of the airway epithelium had similar effects on reducing the actin remodelling observed during infection and attenuating viral entry. Actin projections were associated with NCL interacting with RSV particles resting on apical cilia of the ALIs. We conclude that macropinocytosis-like actin projections protrude through normally protective cilia and mucus layers of stratified airway epithelium that helps present the IGF1R receptor and the NCL coreceptor to RSV particles waiting at the surface.

## Introduction

Respiratory syncytial virus (RSV) infects the respiratory epithelium, primarily ciliated bronchial epithelial cells, in the airways of infants and the elderly [1]. Infection begins when RSV binds to one of its cellular receptors. Many candidate receptors have been previously identified for RSV including heparan sulphate proteoglycans (HSPGs) [2, 3], CX3C chemokine receptor 1 (CX3CR1) [4, 5], epidermal growth factor receptor (EGFR) [6, 7], toll-like receptor 4 (TLR4) [8], intercellular adhesion molecule 1 (ICAM-1) [9], and nucleolin (NCL) [10, 11]. Recently, our lab has identified insulin-like growth factor one receptor (IGF1R) as a receptor for RSV [12]. These receptors have been reported to interact with either the RSV fusion glycoprotein (RSV-F; ICAM-1, IGF1R, EGFR and NCL) or the RSV attachment glycoprotein (RSV-G; CX3CR1 and HSPGs) to coordinate infection. In the context of IGF1R, downstream signalling occurs through a number of pathways following the dimerization of IGF1R and RSV-F: including the phosphoinositide three kinase (PI3K) [13] and mitogen activated protein kinase (MAPK) [14, 15] pathways. Furthermore, IGF1R signals through protein kinase C zeta (PKCζ) to recruit NCL, the RSV coreceptor, to the cell surface [12]. NCL binding to RSV-F then triggers fusion and entry of RSV particles. RSV fusion primarily occurs at the cell surface [16, 17] or in endosomes following endocytosis [6, 7, 12]. In the context of endocytosis, RSV enters host cells via macropinocytosis mediated by PI3K activation [7], which is triggered by RSV binding to growth factor receptors like EGFR and IGF1R [6, 7, 12]. Macropinocytosis is dependent on actin polymerization and thus, interrupting actin dynamics interferes with entry [7].

In this study, we reconcile the different reports on RSV receptors, macropinocytosis, and virus entry to understand how actin, macropinocytosis, and RSV entry receptors interact. Here, we use a bronchial epithelial cell line and air-liquid interface model (ALIs) of the airway epithelium to model RSV entry. ALIs are derived from human donors and express cilia and produce mucus at the air interface that makes for a realistic model of epithelial layers exposed to the lumen of the airway. Our data suggests that RSV reduces the expression of viral receptor on the cell surface following infection. This reduction occurs shortly after infection and corresponds with viral entry into productive cells. Entry and recruitment of NCL were impeded when actin polymerization and PKCζ were inhibited. In fact, macropinocytosis of RSV particles and changes in the actin cytoskeleton associated with viral entry were not observed when IGF1R, PKCζ, or PI3K signalling were abolished. We found that when we added RSV to ALIs, the virus remained on the surface of cilia amongst the mucus layer of the cultures. PI3K and PKCζ activation led to actin protrusions extending through the cilia mucus layer and helped present NCL to virus on the surface. Developing a complete understanding of the mechanism of RSV infection potentiates the development of therapeutics to limit RSV entry and infection.

## Methods

### Cell culture

Primary normal human bronchial epithelial (NHBE) cells (gifted from H. Vliagoftis [University of Alberta] and D.P.) were cultured, and passaged up to a maximum of four times, in Lonza BEGM Bulletkit media (CC-3170) containing retinoic acid. Immortalized human airway epithelial (1HAEo−) cells (a gift from D. Gruenert, University of California) were propagated in minimum essential media (MEM) supplemented with 10 % heat-inactivated FBS. A 1HAEo- *IGF1R^-/-^* CRISPR knockout cell line was generated as described previously [12]. HeLa and A549 cells purchased from America Type Culture Collection (ATCC, CCL2 and CCL185, respectively) were grown in Dulbecco’s Modified Eagle Medium (DMEM), in the presence of l-glutamine, high glucose, sodium bicarbonate and 10 % heat-inactivated FBS. All cell types were grown at 37 °C and 5 % CO_2_ in a humidified incubator. Mycoplasma contamination of cell and RSV stocks were monitored by performing routine screening with the MycoAlert mycoplasma detection kit (Lonza, LT07-118).

### Isolation of human bronchial epithelial cells

A tissue retrieval service (IIAM) provided human lungs not designated for transplant. Dissected airways (main stem bronchus to fourth generation) underwent protease digestion as described [18] to isolate human bronchial epithelial (HBE) cells. Aliquots of cells were stored in liquid nitrogen until use. Each n value corresponds to different individual donors.

### ALI culture of HBE cells

HBE cells were cultured for 72 h using Bronchial Epithelial Growth Medium (BEGM, Lonza), containing 5 % FBS (Life Technologies) before replacing with fresh BEGM lacking FBS every 48 h. Cells were lifted after reaching 90 % confluency before seeding 2.0×10^5^cells per insert onto 1.12 cm^2^, 0.4 -µm pore transwell inserts (Costar) lined with bovine collagen Type I/III (Advanced BioMatrix) and cultured in BEGM for 48 h. Next, BEGM was replaced with basolateral PneumaCult-ALI differentiation medium, with a fresh media change every 48 h, containing 100×supplement, hydrocortisone and heparin (Stemcell Technologies) as well as fluconazole (Sigma-Aldrich) and penicillin/streptomycin (Life Technologies). Cells were washed apically with PBS once a week beginning 2 weeks after seeding to remove excess mucus. Experiments were performed with the cells 5 weeks after initial seeding in transwells.

### Reagents

The following antibodies were used during experimentation: anti-goat IgG H and L (β-galactosidase) (Abcam, ab136712), anti-RSV3 (Novocastra, NCL-RSV3), anti-RSV polyclonal (Abnova, PAB13816), anti-β-actin (Abcam, ab8227), anti-β-tubulin (Sigma-Aldrich, T5201-100UL), anti-phosphorylated (phospho)-p38 MAPK (Thr180/Tyr182) [D3F9] (CST, 4511), anti-SOD-1 [FL-154] (Santa Cruz, sc-11407), anti-phospho-Akt (Thr308) [D25E6] (CST, 13038), anti-phospho-p44/42 MAPK (Erk1/2) (Thr202/Tyr204) [D13.14.4E] (CST, 4370), anti-C23 (NCL) monoclonal [D-6] (Santa Cruz, sc-17826), anti-C23 (NCL) polyclonal [H-250] (Santa Cruz, sc-13057), anti-RSV polyclonal (Meridian, B65860G), anti-phospho-IGF1Rβ Tyr1135 [DA7A8] (CST, 3918), anti-PKCζ [H-1] (Santa Cruz, sc-17781), anti-IGF1R polyclonal (R and D, AF-305-NA), anti-EEA1 [C45B10] (CST, 3288), MUC5AC monoclonal (Sigma, MAB2011) and normal goat control IgG (R and D, AB-108-C). HRP-conjugated light-chain-specific antibodies from Jackson Labs were used for Western blots and Alexa-dye-conjugated secondary antibodies (488, 568 and 647) from Thermo Fisher were used for imaging and flow cytometry.

Chemical inhibitors and activators of cellular processes (targets are listed in square parenthesis) are as follows: PQ401 [IGF1R] 30 µM (Tocris, 2768), U0126 [MEK1/2 (upstream of ERK1/2)] 10 µM (Tocris, 1144), LY294002 [PI3K] 20 µM (Tocris, 1130), PKCζ pseudosubstrate [PKCζ] 3 µM (Tocris, 1791), Antennapedia [PKCζ control] 1.5 µM (Novus, NBP2-29334-5mg), SB203580 [p38 (#1)] 20 µM (Tocris, 1202), BIRB796 (Doramapimod) [p38 (#2)] 10 µM (Selleckchem, S1574), Brefeldin A [inhibits protein transport from ER to golgi] 2 µg ml^−1^ (CST, 9972), Jasplakinolide [stabilizes F-actin] 200 nM (Tocris, 2792), Cytochalasin D [disruptor of actin filament function] 8 µM (Tocris, 1233), CK666 [Actin-Related Protein Complex 2/3 (Arp2/3)] 30 µM (Tocris, 4984), and CK869 [Arp2/3] 40 µM (Tocris, 3950).

### RSV reverse genetics, propagation, and sucrose-density gradient purification

M. E. Peeples (Children’s Research Institute, Columbus) gifted the laboratory-adapted RSV type-A2 strain expressing GFP (rgRSV RW3027). Infectious virus was rescued from full-length rgRSV RW30 cDNA in HeLa cells. Briefly, sub-confluent HeLa cells were transfected with rgRSV RW30 alongside four support plasmids (RSV N, P, L, and M2-1), and T7 RNA polymerase (a gift from B. Lee; Addgene plasmid 65 974, in place of vaccinia virus MVA-T7) using TransIT-HeLa MONSTER (Mirus Bio, MIR 2900). RSV was propagated in HeLa cells after rescue and subsequently collected as cell-free (clarified) RSV-conditioned DMEM with 10 % FBS. To precipitate the RSV, conditioned media was mixed with 10 % polyethylene glycol (PEG) 6000 for 90 min on ice, then pelleted by centrifugation at 4 300 ***g*** at 4 °C for 30 min. Next, the pellets were resuspended in NT buffer (0.15 M NaCl, 0.05 M Tris, pH 7.5) and added to a discontinuous sucrose gradient (35 %, 45 %, 60 % sucrose in NT buffer), as described previously [19]. Following a 4 h ultracentrifuge spin at 217 290 ***g*** at 4 °C, the sucrose-purified RSV band was collected and stored in liquid nitrogen.

### Infectious RSV quantification

Infectious RSV titres were calculated by inoculating confluent 1HAEo− cells with a serial dilution of sucrose-purified virus in MEM containing 10 % heat-inactivated FBS growth medium for 2–4 h at 37 °C, 5 % CO_2_, humidified. Next, cells were washed, fresh growth media was added, and cells were incubated for another 14–16 h before fixation and permeabilization with methanol:acetone (1 : 1). Cells were first blocked with PBS+5 % FBS then stained with goat polyclonal anti-RSV antibody (1 : 1000 dilution), followed by an anti-goat secondary antibody conjugated to β-galactosidase (1 : 2000 dilution). X-Gal substrate (5-bromo-4-chloro-3-indoyl-β-galactopyranoside in PBS containing 3 mM potassium ferricyanide, and 1 mM magnesium chloride) was then added. β-Galactosidase mediated cleavage of X-Gal substrate produces an insoluble blue precipitate indicating RSV infection. Blue cells were manually counted as RSV focus-forming units by light microscopy using the EVOS FL Auto Cell Imaging System (Invitrogen). It is important to note that this technique quantifies infected cells during the first round of RSV infection and requires only viral protein expression to occur rather than completion of the entire viral life cycle as is the case in plaque assays.

To determine the effect of the inhibition of cellular processes on RSV entry, confluent 1HAEo− cells were pre-treated with inhibitor for 1 h, then infected with RSV. Next, cells were acid-washed with 3 M glycine 4 h post-infection to remove virus that had not entered. Finally, cells were washed with media three times, incubated for another 14–16 h, then fixed, stained and counted as described above.

### Viral entry assay

This assay is used to determine the specificity of RSV entry to study the locations and interactions between virus particles and their cellular receptors on the surface of the cell. Cells were synchronously infected with RSV by cooling the cells for 10 min on ice before substituting growth media with media containing RSV (multiplicity of infection of one) for 1 h on ice. Inoculating media was subsequently replaced with fresh, pre-warmed media which constituted time zero. At 4 h later, the cells were acid washed with 3M glycine to remove non-internalized viral particles and fresh media was added. Cells were then incubated at 37 °C for the designated times.

### SDS–PAGE and Western blot

To collect protein lysates, cells were chilled, washed with ice-cold PBS and then lysed with either MOSLB buffer or using the NE-PER (Thermo Fisher, 89842) fractionation kit. Collected protein lysates were heated to 100°C for 10 min with 1×Laemmli sample buffer supplemented with 2.5 % 2-mercaptoethanol as a reducing agent. Samples were loaded on precast Mini-PROTEAN TGX polyacrylamide gradient gels (Biorad) and ran at 150 V for 1 h in Tris-glycine-SDS running buffer. Separated proteins were wet-transferred onto a nitrocellulose membrane at 100 V for 1 h at 4 °C in Tris-glycine transfer buffer supplemented with 10 % methanol. Membranes were washed with Tris-buffered saline containing 0.1 % Tween (TBS-T), blocked with 5 % BSA in TBS-T for 1 h at room temperature. Membranes were probed with primary antibodies, followed by secondary antibodies conjugated to HRP, in TBS-T containing 1 % BSA. Chemifluorescence was generated by adding ECL2 substrate (Pierce pi80196) to blots for 5 min at room temperature, then removed for blots to be imaged. Blots were imaged with a BioRad ChemiDoc MP, detecting HRP using the Cy2 filter and markers using the Cy5 filter. Adobe Photoshop or ImageJ was used to adjust image brightness and contrast.

### Microscopy

The 1HAEo− cells were grown on Labtek II chambered coverslips (Thermo Fisher, 12565338). RSV infections were synchronized by cooling the cells on ice for 15 min before infecting with virus for 1 h on ice before adding fresh media, warmed to 37°C, for the indicated time. Cells were fixed with 4 % paraformaldehyde (PFA) and blocked with PBS+1 % FBS. Cells were stained with primary antibody targeting the indicated proteins for 1 h at room temperature or at 4 °C overnight, and subsequently stained with secondary antibody for 1 h at room temperature. To permeabilize cells (if required), antibody, blocking and wash buffers were supplemented with 0.3 % Triton X-100. ActinGreen 488 ReadyProbes (Thermo Fisher, R37110) and DAPI (0.33 µg ml^−1^ in water) were used to stain actin and cell nuclei, respectively. Cells were stored in Vectashield (Vector Labs, H-1000) at −20 °C to preserve fluorescence. Images were acquired using a spinning disc confocal microscope (Wave FX #2, Quorum Technologies) running Volocity software (PerkinElmer) in the Cell Imaging Centre at the University of Alberta.

ALI-differentiated HBE cells underwent similar infection and staining protocols. Briefly, cells were pre-treated with inhibitors (if applicable) for 1 h at 37 °C before cooling on ice for 20 min. To synchronize infection, cells were incubated for 1 h on ice in media containing RSV and inhibitors. Cells were then either fixed immediately or warmed for 3 h at 37 °C prior to fixing. The ALI-differentiated cultures were permeabilized overnight in PBS+0.3 % Triton X-100 to account for increased thickness relative to a cell monolayer. Cells were then blocked and stained as detailed in the previous paragraph. ALI-differentiated cultures were cut out of the transwell inserts and transferred to microscopy slides for imaging on the Wave FX#2 spinning disc confocal microscope.

Images on the spinning disc confocal microscope were captured using the following excitation and emission parameters: DAPI or Alexa 405: excitation 44 mW, 405 nm, emission 460/50 nm; GFP, ActinGreen or Alexa 488: excitation 50 mW, 491 nm, emission 525/50 nm; Alexa 568: excitation 50 mW, 561 nm, emission 620/60 nm; Alexa 647: excitation 45 mW, 642 nm, emission 700/75 nm. Volocity software was used to analyse images and calculate Pearson’s correlations. To calculate actin variance, a region of interest (ROI) was drawn through the actin cortical cytoskeleton in the XZ plane of the ALI epithelial monolayer. Volocity was used to calculate the variance of the mean fluorescence intensity of actin within the ROI.

### Imaging flow cytometry

The 1HAEo− cells were grown till 95 % confluent under normal conditions. Next, the cells were pre-treated with an inhibitor, when applicable, for 1 h at 37 °C and the concentration of the inhibitor was maintained until cells were detached. Then, as seen above, our viral entry assay was performed. Next, cells were washed with 4 °C PBS before dissociating with a combination of 10 mM EDTA in PBS on ice and scraping. To reduce background signalling, cells were blocked with 5 % FBS in PBS for 30 min on ice. Afterwards, surface NCL was labelled using rabbit polyclonal anti-C23 antibody (1 : 100 dilution) and RSV was stained using the mouse anti-RSV four monoclonal blend (RSV3, 1 : 400 dilution) for 30 min on ice. Secondary staining was performed using a donkey anti-rabbit Alexa 647 antibody (1 : 200 dilution) and a goat anti-mouse Alexa 488 antibody (1 : 800 dilution) for 30 min on ice. Cells were fixed in 4 % PFA before staining with DAPI for 5 min on ice. During data acquisition, at least 100 000 single cells were analysed with the Amnis Mark II ImageStream (405 nm, 488 nm and 642 nm excitation lasers, 60× magnification). Data analysis was performed using IDEAS software (Amnis).

### Imaging flow cytometry data analysis

Imaging flow cytometry data was analysed using IDEAS software. Gate values were pre-set to all unbiased analysis of samples. Single cells were differentiated from beads and doublets/clumps by using forward scatter (FSC) area vs FSC aspect ratio intensity. In-focus single cells were gated using an FSC gradient root-mean-square (RMS) histogram (high FSC gradient RMS). Cells that contained fragmented nuclei were gated out with a DAPI (405 nm) aspect ratio histogram as an additional quality-control step (high DAPI aspect ratio events were gated). A NCL (642 nm) area histogram was used in combination with an intensity histogram to gate non-permeable cells (low nucleolin area/intensity) to account for the fact that NCL is found in lower abundance on the cell surface when compared to the cytoplasmic and nuclear regions. A RSV (488 nm) max pixel intensity histogram (low max pixel cells removed) was used to gate for RSV positive cells. The number of viral particles attached to each cell was counted with a spot count detecting bright spots with the following characteristics: a 4 : 1 spot-to-background ratio, a minimum width of 3 pixels, a maximum area of 250 µm^2^, and a minimum intensity similar to the RSV-positive gate. Cells that were bound with 1–5 viral particles were gated for further analysis. A threshold 80 % mask, which removes the lower 20 % of pixels, was used to detect cell-surface NCL. A combination of a virus spot mask, which was dilated by 2 pixels, and a surface NCL mask was used to detect overlap between NCL and RSV. Cells that exhibited a RSV-NCL patching event were selected using a patching area histogram (removing low patching area) and the NCL intensity within the patching mask was recorded. In summary, the gating is as follows: singlets, in focus, nuclear shape, non-permeable-1, non-permeable-2, RSV positive, 1–5 virus spots, patching positive. Total surface NCL and RSV levels were recorded after the non-permeable-2 gate but before the RSV positive gate.

### Cell viability assay

Cells were treated with the specified chemical inhibitors for 5 h, before acid washing with 3M glycine. The acid was removed by washing thrice with media prior to incubating for an additional 14–16 h. PBS containing 0.3 mg ml^−1^ of thiazolyl blue tetrazolium bromide (MTT, Sigma-Aldrich, M2128-1G) was used to test cell viability. Media was replaced with MTT solution and incubated for 1 h at 37 °C. Afterwards, the MTT solution was removed and the remaining precipitate was solubilized in DMSO for 10 min. Absorbance (560 nm) was detected with a GloMax Explorer plate reader (Promega). The MTT solution was prepared fresh for each experiment.

### Histology

Histology and immunohistochemistry was completed as described in [20]. Briefly, ALIs were fixed in formalin, embedded in paraffin, and sectioned to 4 µm thickness. Following deparaffinization, sections were stained using Alcian Blue 8 GX (Sigma, A3157) and Hematoxylin Gills II (Leica Biosystems, 3801500). Sections were also immunostained using a MUC5AC antibody (Sigma, MAB2011) and DAB Peroxidase Substrate (Vector Laboratories, SK4100) [21]. Sections were then dehydrated and visualized with the Olympus BX51 microscope using a 40× objective and Q Capture Pro 6.0 softwares.

## Results

### IGF1R expression in cell lines is associated with infectability by RSV

**Fig. 1.**
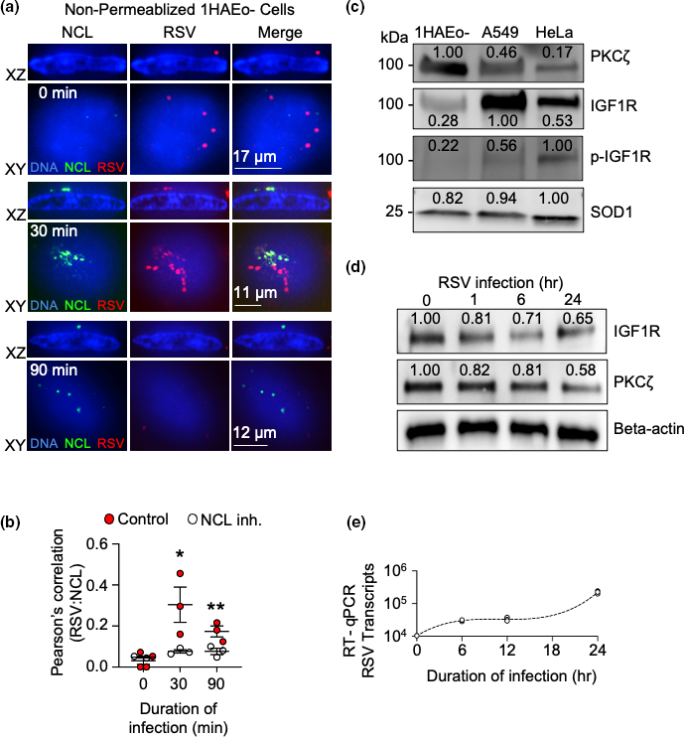
RSV receptor and coreceptor expression on multiple cell lines during a time course of RSV entry. (**a**) Fixed cell confocal microscopy of non-permeabilized RSV infected cells. 1HAEo- bronchial epithelial cells were infected with RSV for 1 h on ice before replacing inoculum with fresh, warm media. Cells were subsequently fixed at the indicated time points following the addition of warm media and were stained for RSV (red), NCL (green) and nuclei (blue). Cells were imaged using a Quorum WaveFX spinning disc confocal microscope. The absence of a permeabilizing detergent allowed for surface staining of antigens whereas the DAPI stain for nucleic acids is lipid permeable allowing visualization of the nucleus. Images are representative of four independent experiments. (**b**) Pearson’s correlation measured the correlation of pixels from RSV staining with NCL staining in control or nucleolin inhibitor treated 1HAEo- cells from three different image stacks at 40× magnification using Volocity. Statistical significance was determined by two way ANOVA with Sidak’s test of significance. *; *P*<0.05, **; *P*<0.01. (**c**) Western blot detection of PKCζ, IGF1R and phospho-IGF1R (p-IGF1R) in uninfected cells; 1HAEo-, A549, and HeLa. SOD1 is a loading control. Densitometry values calculated using ImageJ software relative to the most intense band for each protein. Results show a representative blot from two separate experiments. (**d**) IGF1R and PKCζ levels in 1HAEo- cells were detected by Western blot during a time course of RSV replication. Densitometry values were calculated using ImageJ relative to a beta actin loading control at each respective time point. Results show a representative blot from two separate experiments. (**e**) RSV transcripts (RSV-N gene probe and primers) were detected by RT-qPCR during a time course of RSV infection in a 1HAEo- bronchial epithelial cell line. Experiment was done in triplicate and the mean and standard deviation of the mean are shown. Results are representative of at least three independent experiments.

We previously found that IGF1R is a receptor for RSV [12]. Interaction between IGF1R and RSV-F recruits NCL, an RSV co-receptor [10], to the cell surface via PKCζ signalling. From here, interactions between RSV-F and NCL trigger virion fusion with the host cell plasma membrane. To recapitulate, we stained 1HAEo- human bronchial epithelial cells for RSV and NCL during a viral entry time course (Fig. 1a). Lab adapted RSV-A2 is used for all experiments and will be referred to as RSV for the remainder of the text. We stained the cells prior to permeabilization to image RSV and NCL exclusively at the cell surface. At time 0, we observed RSV particles resting on the cell surface and detected negligible levels of NCL. We observed a peak of NCL at the cell surface 30 min after warming that was significantly reduced 90 min after warming. Peak colocalization signals of NCL and RSV were observed at 30 min post-warming, which are presumably RSV entry fusion reactions (Fig. 1b). By 90 min post-warming, colocalization was decreased. The correlation of RSV:NCL colocalization was significantly higher at each timepoint for control compared to NCL inhibitor treated cells. Areas that showed RSV staining, but not NCL, may represent fusion events that have occurred directly at the cell surface rather than via macropinocytosis (Fig. 1a). In this scenario, the RSV proteins become diffuse as they migrate through the host membrane and represent the dichotomy between direct surface fusion and internalization via endosomes. The absence of visible viral particles at 90 min post-warming suggests that viral particles are completely internalized by this time.

Since IGF1R is a receptor for RSV, it stands to reason that IGF1R expression correlates with increased susceptibility to RSV. To investigate this, we detected the expression of IGF1R using Western blot on a series of uninfected cell lines: 1HAEo-, A549, and HeLa cells (Fig. 1c). These cells are commonly used by our group [12, 19, 22] and others [23–25] to study RSV infection. We have noted that there are differences in the RSV infectious titres between these cell lines, with RSV replicating to a higher titre in HeLas (~200× greater) when compared to A549 or 1HAEo- cells [12]. We detected IGF1R by Western blot and noted that A549 and HeLa cells expressed around four-fold and two-fold more IGF1R than 1HAEo- cells, respectively (Fig. 1c). These changes are compared to a SOD1 loading control in each cell type. Phosphorylated IGF1R at Tyrosine residue 1135 is associated with successful expression of NCL at the cell surface, and with RSV infection [12]. Moreover, we see that IGF1R is phosphorylated in uninfected HeLa cells (Fig. 1c). This constitutively phosphorylated IGF1R may help traffic more NCL to the cell surface independently of IGF1R-ligand interactions. In summary, HeLa cells express more constitutively phosphorylated IGF1R which may potentiate infection by increasing surface NCL levels as previously described [12].

### IGF1R and PKCζ expression are downregulated during RSV replication

Viruses are well known to regulate the expression of receptors at the cell surface. Counterintuitively, the downregulation of viral receptors aids virus infection by enhancing viral release, limiting super infection and restricting host immune responses [26]. Therefore, we aimed to investigate whether IGF1R expression was downregulated during infection with RSV. To this end, we measured expression of IGF1R in 1HAEo- cells over 24 h via Western blot following RSV infection (Fig. 1d). We noted that there was a reduction in IGF1R expression 1 and 6 h after infection. This was accompanied by a gradual reduction of PKCζ band intensity relative to loading controls over 24 h. These reductions match the increase in viral replication in 1HAEo- cells (Fig. 1e). Altogether, these results suggest that RSV causes a gradual reduction of the viral receptor over the course of infection. This reduction corresponds to a decrease in cell signalling through PKCζ.

### Disruption of actin polymerization during RSV entry significantly inhibits RSV infection

**Fig. 2.**
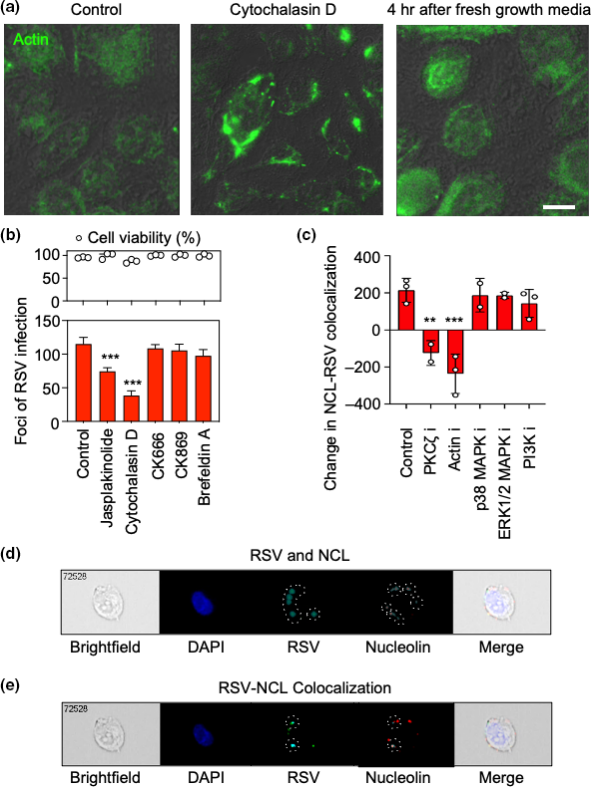
Actin polymerization is required for RSV interaction with NCL during entry. (**a**) 1HAEo- cells were treated with cytochalasin D or DMSO control for 1 h. Cytochalasin D was also removed from the cells and replaced with normal growth media for 4 h. Subsequently, all cells were fixed and stained for actin. Scale bars=10 µm. (**b**) Cells were inoculated with RSV in the presence of inhibitors for 4 h during viral entry, washed with normal growth media, and RSV infection (left y-axis) was detected and enumerated the next day by anti-RSV immunostaining. Subsequently, the cell viability was determined by MTT assay and reported as a percentage of the control DMSO treated cells (right y-axis). Significance was determined by ANOVA with Bonferroni test of significance. ***; *P*<0.0001. Data presented are the result of three independent experiments. (**c**) 1HAEo- cells treated with the specified inhibitors were inoculated with RSV for 0 and 60 min before they were fixed and stained for RSV and NCL. RSV interaction with cell surface NCL was quantified by Imagestream imaging flow cytometry. The change in RSV-NCL co-localization between 0 and 60 min is shown. Statistically analysed by one-way ANOVA and Tukey’s post-hoc test, comparing each treatment to the control. **; *P*<0.01, ***; *P*<0.005. Results are the means from at least two independent experiments. Cytochalasin D was used as the actin polymerization inhibitor. (**d and e**) Gating strategy for Imagestream Analysis in (**c**). RSV and NCL were detected on 1HAEo- cells using Imagestream software and (**e**) RSV colocalization with cell surface NCL was quantified using user defined intensity and size thresholds for RSV and NCL staining.

In a paper published by Krzyzaniak *et al*. [7] they showed that actin rearrangement was necessary for optimal RSV entry and infection of HeLa cells. Before we could elucidate the effects of actin disruption on RSV entry and infection, we tracked the stability of the cell cytoskeleton before, during, and after disrupting actin polymerization with cytochalasin D treatment (Fig. 2a). Here, we noted that cytochalasin D treatment resulted in dense intracellular actin foci, presumably by changing the disruption and retraction of actin filaments into bundles. When cytochalasin D was replaced with normal growth media, we noted that the appearance of the actin stress fibres resembled those observed in the control treatment. This suggests that the actin cytoskeleton in drug treated cells was disrupted only when cytochalasin D was present, and that cytochalasin D could temporarily perturb the actin cytoskeleton during virus adsorption and internalization.

To study the effects of actin disruption on RSV entry and infection, we pretreated 1HAEo- cells with a variety of drugs that affect actin polymerization for 1 h (Fig. 2b). The cells were subsequently infected with RSV in the presence of inhibitors and infection was allowed to proceed for 4 h before removing virions that had not yet been internalized. Since actin disruption is reversible (Fig. 2a), this ensured that the actin cytoskeleton was disrupted exclusively during RSV entry. We noted that Jasplakinolide, an actin stabilizing drug, and cytochalasin D, an actin disrupting drug, were most effective at reducing RSV titres (Fig. 2b). Cytochalasin D exhibited the greatest inhibition and resulted in an approximate ten-fold reduction in viral titres. Conversely, treatment with CK666 and CK869, which inhibit the actin-related protein complex 2/3 (Arp2/3), as well as brefeldin A, which perturbs Golgi trafficking, had minimal effect on RSV titres. All of the tested compounds did not significantly impact cell viability. While these data show that disrupting actin dynamics impacts viral titres, they do not pinpoint the exact step of RSV replication that is impacted.

### Disruption of actin reduces the interaction of RSV with its coreceptor NCL

Hovanessian *et al.* have previously shown that cell surface NCL expression is dependent upon an intact cortical actin cytoskeleton [27]. To better understand how actin disruption impacts RSV infection, we explored the role of actin in the interaction between RSV and its coreceptor, NCL. We postulated that disruption of actin polymerization would impact RSV and NCL interactions during entry. Using the same cell surface staining method as described above in Fig. 1a, we quantified the RSV-NCL interactions at the cell surface of 1HAEo- cells using imaging flow cytometry (Fig. 2c). The actin inhibitor cytochalasin D resulted in a significant reduction in NCL-RSV patching events. These data suggested to us that actin rearrangement was necessary for NCL trafficking to the cell surface. Furthermore, inhibiting PKCζ, but not p38, ERK1/2 or PI3K, resulted in a significant decrease in NCL and RSV colocalization suggesting that PKCζ signalling plays a dominant role in trafficking NCL to the cell surface. Given that RSV signals through PKCζ after binding to IGF1R, it is possible that the interaction between RSV and IGF1R promotes actin remodelling that permits RSV entry [12].

### RSV causes actin remodelling and enters through macropinocytosis that is significantly reduced in *IGF1R^–/–^* cells

**Fig. 3.**
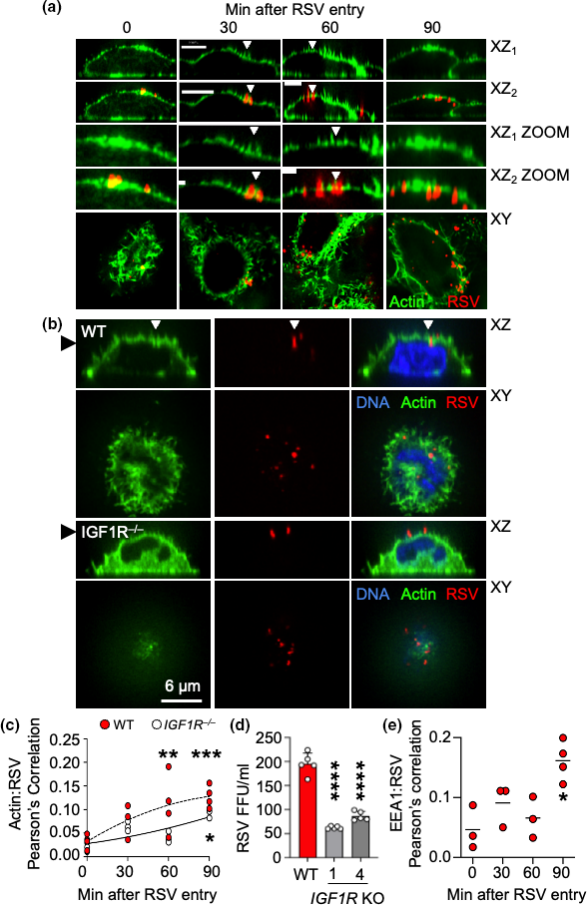
Cortical actin cytoskeleton remodelling occurs during RSV entry followed by RSV entry into EEA1 early endosomes. (**a**) 1HAEo- cells were infected with RSV for the indicated duration, stained for RSV (red) and actin (green) and imaged by confocal microscopy. Arrowheads show micropinocytosis-like actin projections forming around RSV particles. Scale bars are 8 µm. Images are representative of four independent experiments. (**b**) WT and *IGF1R^–/–^* CRISPR KO 1HAEo- cells were infected and stained as described in (**a**) above, with the addition of a DAPI stain for DNA, and fixed 90 min after infection. White arrowhead shows RSV particle entering across the cortical actin cytoskeleton. Black arrowhead denotes the image in the XZ stack that is shown as XY. Images are representative of three independent experiments. (**c**) RSV foci of infection in two different *IGF1R-/-* CRISPR clones one and four. Significance was determined by ANOVA with Dunnett’s test. ****; *P*<0.0001. Experiments were repeated twice with similar results. (**d**) Pearson’s correlation in Volocity imaging software was used to calculate the colocalization between RSV and actin during RSV entry into WT (red filled circles) and *IGF1R^–/–^* CRISPR KO 1HAEo- cells (empty circles). This was from the experiment used to produce the images shown in (**b**). (**e**) Pearson’s correlation was used to quantify the interaction of RSV with early endosome antigen 1 (EEA1). One-way ANOVA with Tukey’s test determined significance. *; *P*<0.05, **; *P*<0.01. Each data point represents an independent experiment of the Pearson’s correlation of at least ten cells per experiment.

It was previously reported that EGFR signalling through PI3K resulted in entry of RSV into HeLa cells via macropinocytosis [7]. We first investigated whether we could observe changes in the cortical actin cytoskeleton of 1HAEo- cells during infection with RSV (Fig. 3a). Here, we noted that the cortical actin cytoskeleton stain was concentrated beneath RSV particles seated on the plasma membrane. A hallmark of macropinocytosis is the formation of finger-like actin projections around virus particles and we observed these projections around viral particles at 30 and 60 min after RSV entry. We observed gaps forming in the cortical actin cytoskeleton beneath RSV particles 60 and 90 min after RSV entry. We observed RSV particles descending beneath the plane of the actin cortical cytoskeleton at 90 min.

Given that IGF1R similarly signals through PI3K [13], we postulated that IGF1R may also play a role in macropinocytosis of RSV. To investigate, we ran a time course of RSV entry in *IGF1R^–/–^* cells (Fig. 3b). WT 1HAEo- cells exhibited gaps in the actin cytoskeleton and protrusions surrounding viral particles at 90 min post-infection. RSV particles were observed under the actin cytoskeleton as well, suggesting that internalization of virions was largely completed. Conversely, neither actin-protrusions nor gaps in the cytoskeleton were observed during infection of *IGF1R^–/–^* cells with RSV. Furthermore, RSV remained at the surface of the *IGF1R^–/–^* host cell even 90 min post-infection which suggests that internalization and actin rearrangement was severely impacted in mutant cells. We quantified the progress of entry in this experiment by determining the colocalization of RSV stain with actin stain using Pearson’s correlation (Fig. 3c). There was a significant increase in correlation between actin and RSV at 60 min post-infection in WT 1HAEo- cells. Correlation between actin and RSV did not reach significance until 90 min post-infection in *IGF1R^–/–^* cells. RSV titres were severely reduced, but not completely abrogated, in *IGF1R^–/–^* cells (Fig. 3d). This suggests that IGF1R signalling is an important but not the sole mediator of RSV entry. Since there are many other reported receptors for RSV, it is possible that another receptor promotes actin cytoskeletal changes promoting RSV entry in the absence of IGF1R. Overall, this finding supports the hypothesis that IGF1R signalling is involved in actin remodelling during RSV entry as well as trafficking of the RSV coreceptor NCL to the cell surface.

Krzyzaniak also showed that RSV enters Rab5-positive early endosomes after crossing the cortical actin cytoskeleton [7]. To confirm these results, we used early endosome antigen-1 (EEA1) to investigate whether RSV is incorporated into early endosomes during entry. We observed the greatest amount of RSV colocalization with EEA1 90 min after infection which aligns with the previous report (Fig. 3e).

### IGF1R mediates NCL trafficking to RSV particles during entry in air-liquid interface cultures

**Fig. 4.**
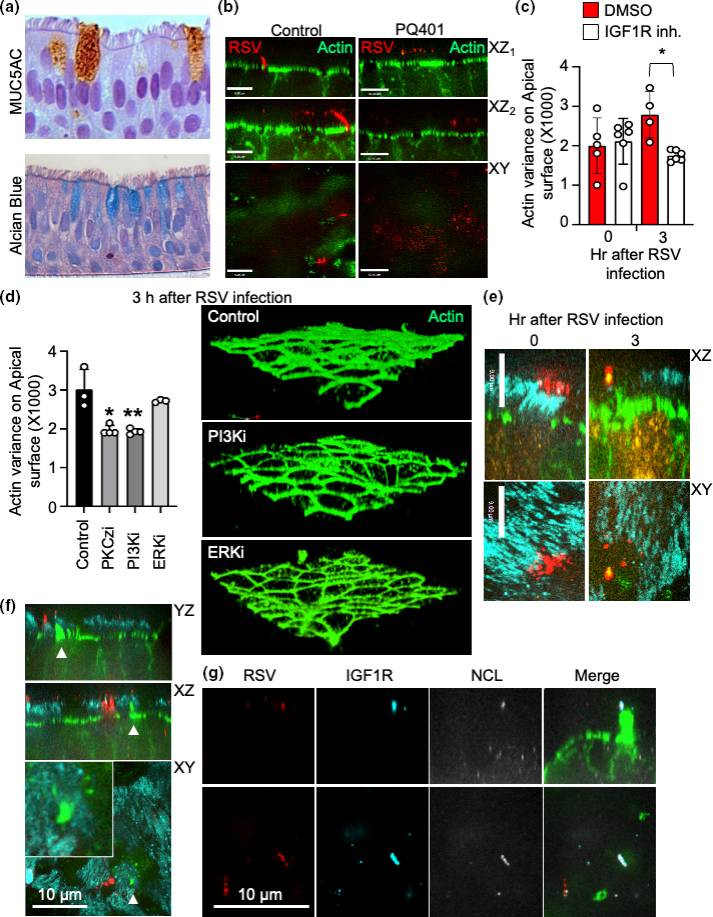
PI3-kinase signalling is required for cortical actin cytoskeleton remodelling in stratified RSV infected air-liquid interface (ALI) cultures. (**a**) ALI cultures were fixed and processed for histology as described. Mucin production was detected in goblet cells using an anti-MUC5AC antibody (top; brown) or with alcian blue (bottom; blue). (**b**) Immunofluorescence images of ALI cultures infected with RSV in the presence or absence of IGF1R inhibitor PQ401. ALI cultures were treated with or without IGF1R inhibitor for 1 h before infecting with RSV. Afterwards, cellular actin (green) and RSV (red) were labelled. Scale bars=9 µm for control and 10 µm for PQ401. Images are representative of two separate donors. (**c**) Actin variance was quantified for DMSO and PQ401 treated ALIs by defining the region of interest (ROI) above the actin cortical cytoskeleton in the XZ plane of the cortical cytoskeleton. Actin staining variance of the mean fluorescence intensity in the ROI was calculated using Volocity software. Each point denotes the variance from the ROI in one image stack from one donor and data are representative of two experiments with two donors each. (**d**) Representative immunofluorescence images and the corresponding actin variance of ALI cultures treated with or without PI3K or ERK inhibitor before infecting with RSV for 3 h. Actin variance was measured using the same method as (**c**). Data are representative of two experiments with two donors each. (**e-f**) Immunofluorescence images of ALI cultures infected with RSV for 0 (left) or 3 h (right) (figure E) or for 3 h (figure F). Images depict β-tubulin (cyan), nucleolin (yellow), RSV (red), and actin (green). (**g**) Immunofluorescence images of ALI cultures infected with RSV. Images depict RSV (red), IGF1R (cyan), nucleolin (white), and actin (green). The data shown in figures E-G are representative of two repeats using ALI made from three different adult donors.

We generated air-liquid interface (ALI) cultures of HBE cells to investigate cytoskeleton restructuring. We tested appropriate differentiation of the ALI cultures by detecting mucins with alcian blue and MUC5AC (Fig. 4a). Mucins were readily detected with both stains suggesting that the ALIs had properly differentiated and could be used as a more accurate reflection of a physiological infection. Next, we infected ALI cultures in the presence or absence of PQ401, an IGF1R inhibitor, to investigate its effect on RSV entry (Fig. 4b). In the absence of IGF1R inhibitor, we observed an influx of RSV entering through apical gaps in the cortical actin cytoskeleton of ALI cultures. Conversely, this influx was absent in the presence of the IGF1R inhibitor. Additionally, we observed more RSV particles adhered to the apical surface of the cells in the presence of IGF1R inhibitor when compared to the untreated cells. Next, we calculated actin variance in our ALI model by measuring the total intensity of actin fluorescence above the apical surface of the ALIs. Interestingly, we observed decreased proportions of actin present above the cortical cytoskeleton in the presence of IGF1R inhibitor (Fig. 4c). This suggests that IGF1R is important in actin remodelling and mediating internalization of viral particles in ALI cultures.

We then wanted to determine which molecules downstream of IGF1R mediate viral entry. To do so, we used chemical inhibitors to interfere with multiple arms of the IGF1R signalling pathway and investigated their impact on the actin cytoskeleton (Fig. 4d-g). Inhibition of PKCζ and PI3K, but not ERK, reduced actin variance measured above the apical actin cytoskeleton after infection with RSV (Fig. 4d). Reduced actin variance could be observed using immunofluorescence microscopy of ALI cultures. In untreated samples, the apical cytoskeleton was perturbed and hallmarks of macropinocytosis could be observed. These features were absent when ALI cultures were treated with PI3K inhibitors. No differences in actin variance were observed immediately following virus adsorption (Fig. S1, available in the online version of this article) suggesting that differences in actin structure arose due to alterations in signalling rather than the presence of the inhibitors themselves.

### RSV and IGF1R signalling results in the formation of actin podia at sites of entry

During immunofluorescence experiments, we observed the formation of actin tail structures directed toward bound virus particles (Fig. 4e). These structures were only observed after NCL was recruited to the cell surface and not during the initial binding of virus. These actin structures seemed to invade the ciliary layer that RSV initially bound to during viral infection. Larger podia-like structures could be seen migrating towards virus present at the ciliary surface (Fig. 4f-g; Figs S2-3). These structures were observed branching off the apical cytoskeleton towards a subset of viral particles bound to the ciliary surface. Additionally, two viral receptors, IGF1R and NCL, can be seen on these actin projections colocalizing with RSV particles (Fig. 4g). These structures are largely present when NCL is bound to viral particles suggesting that they form either as a mechanism to shuttle NCL towards bound virus or after virus is bound to NCL at the cell surface.

## Discussion

The mucus and cilia of the bronchial epithelium are a thick physical barrier to virus infection. As such, viruses must find a workaround to be able to productively infect bronchial cells. Here, we show that RSV induces actin rearrangement via PI3K to trigger macropinocytosis. Our work suggests that this remodelling of the actin cytoskeleton enables the plasma membrane and receptors to be pushed up toward RSV particles, through these physical barriers.

We noted that RSV infection reduced the expression of IGF1R and PKCζ in 1HAEo- cells over a 24 h period after RSV entry (Fig. 1d). This reduction in the viral receptor was observed just after receptor call-up (Fig. 1a) and viral entry (Fig. 3a) occurred. Since downregulation of IGF1R occurs promptly after infection, this may represent a mechanism used by RSV to restrict super-infection of a single cell and promote the infection of neighbouring, uninfected cells that have unimpeded expression of the viral receptor. Downregulation of viral receptors following a productive infection is a common theme among viruses. For example, HIV has been shown to downregulate CD4, the primary entry receptor, during infection [28, 29]. ACE2 is also downregulated during SARS-CoV-2 infection [30]. It is conceivable that RSV has evolved a similar mechanism to facilitate a successful infection of the host.

Disturbing actin polymerization significantly reduces productive infection of host cells with RSV [7]. We indeed found that both cytochalasin D and jasplakinolide treatment significantly reduced RSV titres (Fig. 2b). Interrupting the ARP2/3 complex, which regulates actin assembly by nucleating filaments [31], had little effect on viral tires. Mehedi *et al.* [32] found that siRNA knockdown and CK666 inhibition of Arp2 had no effect on RSV entry but instead modulated the actin cytoskeleton to reduce viral egress. Our method of quantifying viral titres detects entry and production of viral progeny. Therefore, treatments that impede viral egress would not correspond with a reduction in viral titre. Overall, our results support the findings of Mehedi *et al.* [32] and act as a control to confirm the specificity of our entry assay.

During infection with RSV, we observed changes to the cortical actin cytoskeleton characterized by protrusions surrounding viral particles (Fig. 3a). These protrusions resemble the formation of a macropinocytic cup [33] and the timing of these events support the finding that RSV is internalized via macropinocytosis [7]. Viral particles correlated with EEA1, a marker of early endosomes, following actin rearrangement which further supports the evidence that virus is incorporated into early endosomes following macropinocytosis [7]. However, EEA1 and RSV show limited correlation even at 90 min post-infection which implies that RSV may favour fusion at the cell surface over fusion following internalization. Hallmarks of macropinocytosis could not be detected in *IGF1R^–/–^* cells suggesting that signalling downstream of IGF1R facilitates viral entry through this route. While ligand-induced macropinocytosis has not been observed with IGF1R, it has been with the closely related EGFR [34–36]. Therefore, it is reasonable to presume that IGF1R signalling is capable of inducing macropinocytosis when bound by ligands. This theory is supported by the finding that, during physiological signalling, IGF1R expression in filopodia is reduced following interaction with ligand [37]. This process is also dependent on actin and signalling factors downstream of IGF1R, such as PI3K and MAPK. Our results mirror these findings, which demonstrate a lack of podia and reduced actin variance during PI3K and IGF1R inhibition (Fig. 4). We postulate that RSV is mimicking a ligand of IGF1R as a way to take advantage of host actin remodelling pathways. As IGF1R is bound by RSV, downstream signalling by factors such as PI3K are activated, which in turn triggers actin remodelling to help facilitate entry.

Altogether, we have shown that RSV-induced cytoskeleton rearrangements during the entry process are mediated by IGF1R. More specifically, actin restructuring was abrogated when downstream PKCζ and PI3K, but not ERK, signalling was inhibited. However, inhibition of IGF1R does not completely restrict infection with RSV [12] meaning that other viral receptors may play a role in mediating viral entry. For example, TLR4 is a protein that is known to interact with RSV-F and signal through PI3K [38–40]. Therefore, it is worthwhile to investigate the role that TLR4 signalling plays in inducing cytoskeleton rearrangements that culminate in RSV internalization. Another avenue of exploration lies in the findings that RSV-F undergoes cleavage to different extents between viral subtypes [41]. Specifically, RSV-A retains the p27 subunit to higher proportions than RSV-B which stabilizes the pre-fusion conformation of RSV-F. It would be interesting to determine if the differences in RSV-F stability between the two viral subtypes leads to a preference in infection via direct surface fusion or endocytosis. Overall, our paper provides important insight into the process surrounding RSV entry. A complete understanding of the entry process may facilitate the development of therapeutics that restrict viral replication and stem the burden of disease imposed by RSV.

## Supplementary Data

Supplementary material 1

## References

[1] Griffiths C, Drews SJ, Marchant DJ (2017). Respiratory syncytial virus: Infection, detection, and new options for prevention and treatment. Clin Microbiol Rev.

[2] Feldman SA, Audet S, Beeler JA (2000). The fusion glycoprotein of human respiratory syncytial virus facilitates virus attachment and infectivity via an interaction with cellular heparan sulfate. J Virol.

[3] Krusat T, Streckert HJ (1997). Heparin-dependent attachment of respiratory syncytial virus (RSV) to host cells. Arch Virol.

[4] Johnson SM, McNally BA, Ioannidis I, Flano E, Teng MN (2015). Respiratory syncytial virus uses CX3CR1 as a receptor on primary human airway epithelial cultures. PLoS Pathog.

[5] Chirkova T, Lin S, Oomens AGP, Gaston KA, Boyoglu-Barnum S (2015). CX3CR1 is an important surface molecule for respiratory syncytial virus infection in human airway epithelial cells. J Gen Virol.

[6] Currier MG, Lee S, Stobart CC, Hotard AL, Villenave R (2016). EGFR interacts with the fusion protein of respiratory syncytial virus strain 2-20 and mediates infection and mucin expression. PLoS Pathog.

[7] Krzyzaniak MA, Zumstein MT, Gerez JA, Picotti P, Helenius A (2013). Host cell entry of respiratory syncytial virus involves macropinocytosis followed by proteolytic activation of the F protein. PLoS Pathog.

[8] Kurt-Jones EA, Popova L, Kwinn L, Haynes LM, Jones LP (2000). Pattern recognition receptors TLR4 and CD14 mediate response to respiratory syncytial virus. Nat Immunol.

[9] Behera AK, Matsuse H, Kumar M, Kong X, Lockey RF (2001). Blocking intercellular adhesion molecule-1 on human epithelial cells decreases respiratory syncytial virus infection. Biochem Biophys Res Commun.

[10] Tayyari F, Marchant D, Moraes TJ, Duan W, Mastrangelo P (2011). Identification of nucleolin as a cellular receptor for human respiratory syncytial virus. Nat Med.

[11] Holguera J, Villar E, Muñoz-Barroso I (2014). Identification of cellular proteins that interact with Newcastle Disease Virus and human Respiratory Syncytial Virus by a two-dimensional virus overlay protein binding assay (VOPBA). Virus Res.

[12] Griffiths CD, Bilawchuk LM, McDonough JE, Jamieson KC, Elawar F (2020). Publisher Correction: IGF1R is an entry receptor for respiratory syncytial virus. Nature.

[13] Gusscott S, Jenkins CE, Lam SH, Giambra V, Pollak M (2016). IGF1R Derived PI3K/AKT signaling maintains growth in a subset of human T-cell acute lymphoblastic leukemias. PLoS One.

[14] Hua H, Kong Q, Yin J, Zhang J, Jiang Y (2020). Insulin-like growth factor receptor signaling in tumorigenesis and drug resistance: a challenge for cancer therapy. J Hematol Oncol.

[15] Peruzzi F, Prisco M, Dews M, Salomoni P, Grassilli E (1999). Multiple signaling pathways of the insulin-like growth factor 1 receptor in protection from apoptosis. Mol Cell Biol.

[16] Alonas E, Lifland AW, Gudheti M, Vanover D, Jung J (2014). Combining single RNA sensitive probes with subdiffraction-limited and live-cell imaging enables the characterization of virus dynamics in cells. ACS Nano.

[17] San-Juan-Vergara H, Sampayo-Escobar V, Reyes N, Cha B, Pacheco-Lugo L (2012). Cholesterol-rich microdomains as docking platforms for respiratory syncytial virus in normal human bronchial epithelial cells. J Virol.

[18] Churchill L, Chilton FH, Resau JH, Bascom R, Hubbard WC (1989). Cyclooxygenase metabolism of endogenous arachidonic acid by cultured human tracheal epithelial cells. Am Rev Respir Dis.

[19] Bilawchuk LM, Griffiths CD, Jensen LD, Elawar F, Marchant DJ (2017). The susceptibilities of respiratory syncytial virus to nucleolin receptor blocking and antibody neutralization are dependent upon the method of virus purification. Viruses.

[20] Jamieson KC, Wiehler S, Michi AN, Proud D (2020). Rhinovirus induces basolateral release of IL-17C in highly differentiated airway epithelial cells. Front Cell Infect Microbiol.

[21] Michi AN, Proud D (2021). A toolbox for studying respiratory viral infections using air-liquid interface cultures of human airway epithelial cells. Am J Physiol Lung Cell Mol Physiol.

[22] Elawar F, Griffiths CD, Zhu D, Bilawchuk LM, Jensen LD (2017). A virological and phylogenetic analysis of the emergence of new clades of respiratory syncytial virus. Sci Rep.

[23] Hillyer P, Shepard R, Uehling M, Krenz M, Sheikh F (2018). Differential responses by human respiratory epithelial cell lines to respiratory syncytial virus reflect distinct patterns of infection control. J Virol.

[24] Li Y, Dinwiddie DL, Harrod KS, Jiang Y, Kim KC (2010). Anti-inflammatory effect of MUC1 during respiratory syncytial virus infection of lung epithelial cells in vitro. Am J Physiol Lung Cell Mol Physiol.

[25] Rajan A, Piedra F-A, Aideyan L, McBride T, Robertson M (2022). Multiple respiratory syncytial virus (RSV) strains infecting HEp-2 and A549 cells reveal cell line-dependent differences in resistance to RSV infection. J Virol.

[26] Firsching R, Buchholz CJ, Schneider U, Cattaneo R, ter Meulen V (1999). Measles virus spread by cell-cell contacts: uncoupling of contact-mediated receptor (CD46) downregulation from virus uptake. J Virol.

[27] Hovanessian AG, Puvion-Dutilleul F, Nisole S, Svab J, Perret E (2000). The cell-surface-expressed nucleolin is associated with the actin cytoskeleton. Exp Cell Res.

[28] Jin YJ, Cai CY, Zhang X, Zhang HT, Hirst JA (2005). HIV Nef-mediated CD4 down-regulation is adaptor protein complex 2 dependent. J Immunol.

[29] Stove V, Van de Walle I, Naessens E, Coene E, Stove C (2005). Human immunodeficiency virus Nef induces rapid internalization of the T-cell coreceptor CD8alphabeta. J Virol.

[30] Lei Y, Zhang J, Schiavon CR, He M, Chen L (2021). SARS-CoV-2 spike protein impairs endothelial function via downregulation of ACE 2. Circ Res.

[31] Paluck A, Osan J, Hollingsworth L, Talukdar SN, Saegh AA (2021). Role of ARP2/3 complex-driven actin polymerization in RSV infection. Pathogens.

[32] Mehedi M, McCarty T, Martin SE, Le Nouën C, Buehler E (2016). Actin-related protein 2 (ARP2) and virus-induced filopodia facilitate human respiratory syncytial virus spread. PLoS Pathog.

[33] Jin J, Shen Y, Zhang B, Deng R, Huang D (2018). In situ exploration of characteristics of macropinocytosis and size range of internalized substances in cells by 3D-structured illumination microscopy. Int J Nanomedicine.

[34] Haigler HT, McKanna JA, Cohen S (1979). Rapid stimulation of pinocytosis in human carcinoma cells A-431 by epidermal growth factor. J Cell Biol.

[35] Sorkin A, Goh LK (2008). Endocytosis and intracellular trafficking of ErbBs. Exp Cell Res.

[36] Yamazaki T, Zaal K, Hailey D, Presley J, Lippincott-Schwartz J (2002). Role of Grb2 in EGF-stimulated EGFR internalization. J Cell Sci.

[37] Krndija D, Fairhead M (2019). IGF1R undergoes active and directed centripetal transport on filopodia upon receptor activation. Biochem J.

[38] Laird MHW, Rhee SH, Perkins DJ, Medvedev AE, Piao W (2009). TLR4/MyD88/PI3K interactions regulate TLR4 signaling. J Leukoc Biol.

[39] Marchant D, Singhera GK, Utokaparch S, Hackett TL, Boyd JH (2010). Toll-like receptor 4-mediated activation of p38 mitogen-activated protein kinase is a determinant of respiratory virus entry and tropism. J Virol.

[40] Rallabhandi P, Phillips RL, Boukhvalova MS, Pletneva LM, Shirey KA (2012). Respiratory syncytial virus fusion protein-induced toll-like receptor 4 (TLR4) signaling is inhibited by the TLR4 antagonists *Rhodobacter sphaeroides* lipopolysaccharide and eritoran (E5564) and requires direct interaction with MD-2. mBio.

[41] Rezende W, Ye X, Angelo LS, Carisey AF, Avadhanula V (2023). The efficiency of P27 cleavage during in vitro respiratory syncytial virus (RSV) infection is cell line and RSV subtype dependent. J Virol.

