# Supplementary material for: Actin cytoskeleton remodeling disrupts physical barriers to infection and presents entry receptors to respiratory syncytial virus
Source: J Gen Virol. 2023 Nov 28;104(11):001923. doi: 10.1099/jgv.0.001923 (PMC10768689; doi:10.1099/jgv.0.001923)
Supplement: Supplementary material 1 [file jgv-104-1923-s001.pdf]

Supplementary Figure 1

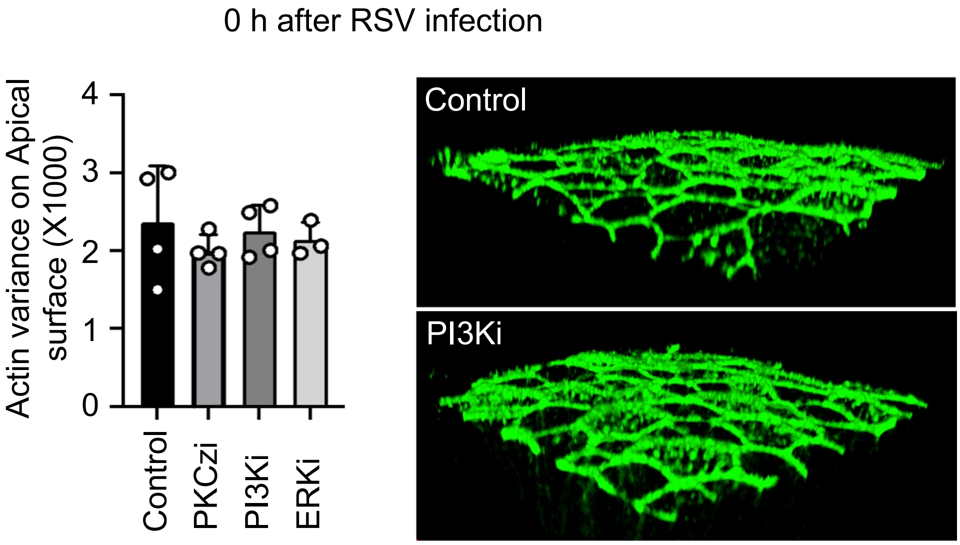

Supplementary Figure 1. The actin cytoskeleton is not perturbed immediately after addition of PKCz, ERK and PI3K inhibitors. Immunofluorescence images and the corresponding actin variance of ALI cultures treated with or without PI3K inhibitors before inoculating with RSV and immediately fixing. No variation can be detected in the actin cytoskeleton.

Supplementary Figure 2

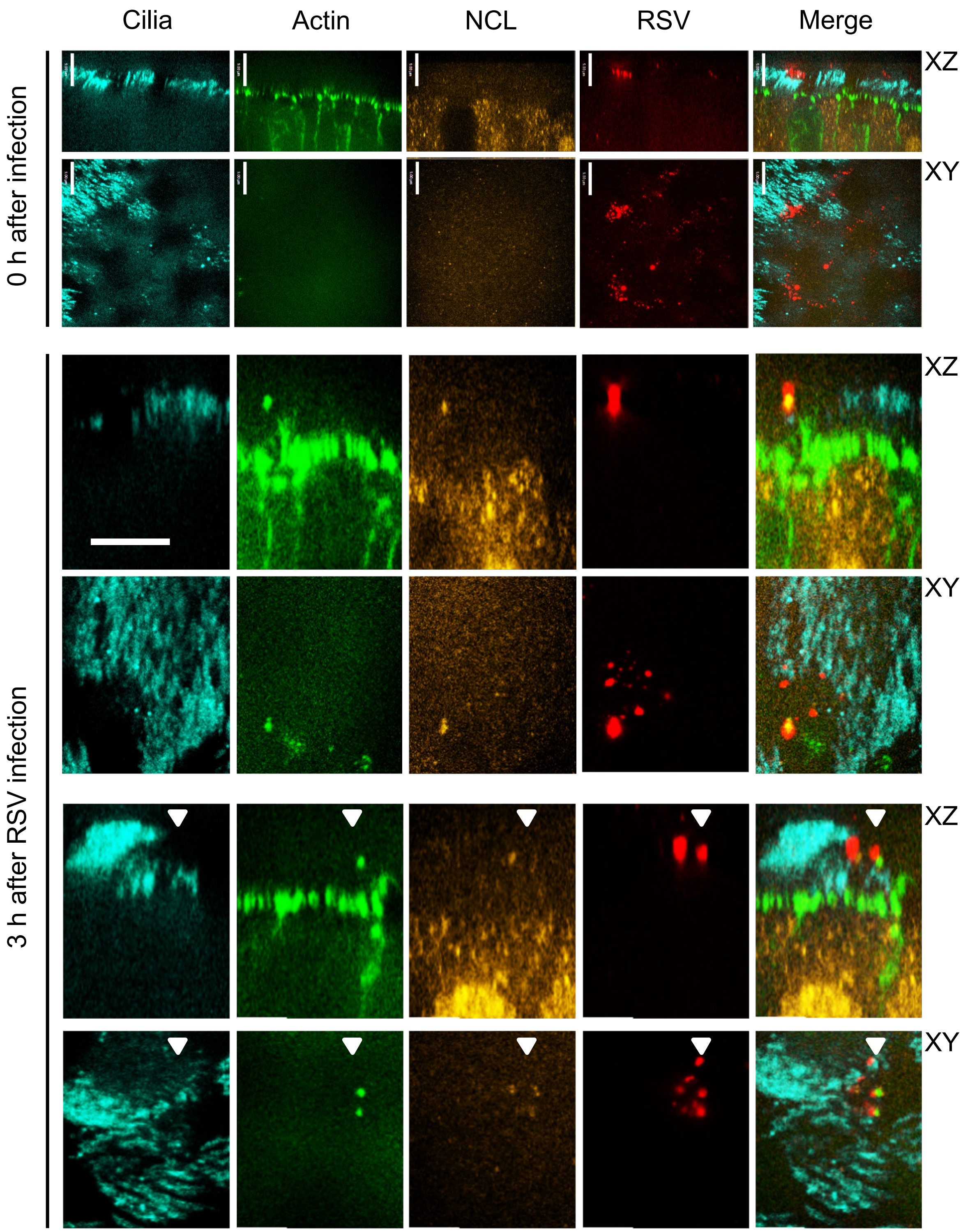

Supplementary Figure 2. Actin podia-like structures migrate towards the RSV/NCL complex. ALI cultures were infected with RSV for 0 hours (top 2 rows) or for 3 hours (bottom 4 rows) before fixing and processing for immunofluorescence microscopy. Images depict β-tubulin (cyan), nucleolin (yellow), RSV (red), and actin (green) and show the individual channels of the micrograph present in figure 4E. Scale bars = 9 µm.

Supplementary Figure 3

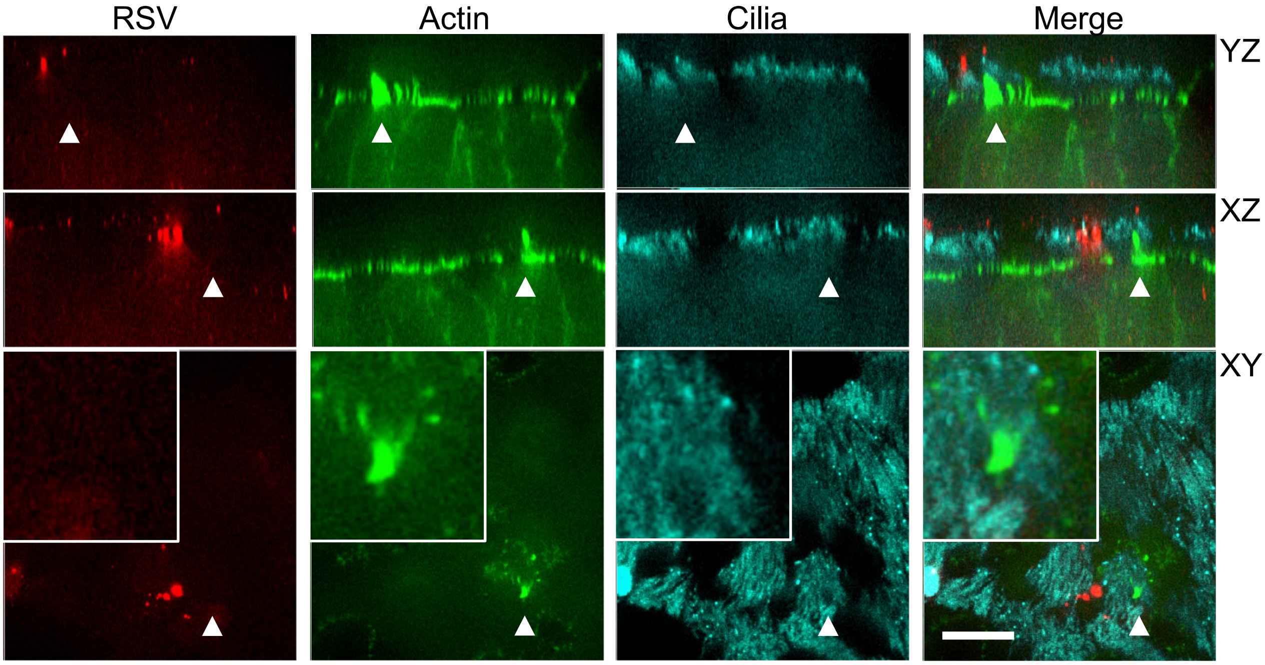

Supplementary Figure 3. Actin podia-like structures are present near adsorbed viral particles. Immunofluorescence images of ALI cultures infected with RSV for 3 hours prior to fixation. Images depict β-tubulin (cyan), RSV (red), and actin (green) and show the individual channels of the micrograph present in figure 4F. Scale bars = 10μm.
